# Supplementary material for: Hybrid Models and Biological Model Reduction with PyDSTool
Source: PLoS Comput Biol. 2012 Aug 9;8(8):e1002628. doi: 10.1371/journal.pcbi.1002628 (PMC3415397; doi:10.1371/journal.pcbi.1002628)
Supplement: Text S4 — Complete source code for the PyDSTool package (version 0.88.120504). Includes API documentation and help files linking to web pages. This file is identical to the current public release on Sourceforge.net. (ZIP) [file pcbi.1002628.s004.zip › PyDSTool/html/PyDSTool.PyCont.TestFunc.DiscreteMap-class.html]

xml version="1.0" encoding="ascii"?


PyDSTool.PyCont.TestFunc.DiscreteMap


| Home | Trees | Indices | Help | PyDSTool |
| --- | --- | --- | --- | --- |

Package PyDSTool ::
Package PyCont ::
Module TestFunc ::
Class DiscreteMap

[hide private]

[frames] | no frames]

# Class DiscreteMap

source code

```
object --+    
         |    
  Function --+
             |
            DiscreteMap
```

---

```
Turns a function into a map composed with itself period times.  Chain rule gives jacobian.
Note that F: R^n+m --> R^n, where m is the number of free parameters.  Thus, we transform to
G: R^n+m --> R^n+m given by G(x,p) = [F(x,p), p], where x is state variable and p parameters.
This gives

    DG = [ DF_x DF_p ]
         [ 0    I    ]

Chain rule on F gives DF^n = DF(F^n-1)*DF(F^n-2)*...*DF(F)*DF(X).  Chain rule on G gives
the same thing as F, and when you keep track of the upper left and upper right blocks of DG composed
with itself, you arrive at

    DF^n_x = DF_x(F^n-1)*DF_x(F^n-2)*...*DF_x(F)*DF_x(X)
    DF^n_a = DF_x(F^n-1)*DF_a(F^n-2) + DF_a(F^n-1) (defined recursively with F^0 = X, F^-1 = 0)
```


|  |  |
| --- | --- |
| Instance Methods | [hide private] |

|  |  |
| --- | --- |
| \_\_init\_\_(self, F, period=1)  x.\_\_init\_\_(...) initializes x; see x.\_\_class\_\_.\_\_doc\_\_ for signature | source code |

|  |  |
| --- | --- |
| func(self, X) | source code |

|  |  |
| --- | --- |
| diff(self, X, ind=None) | source code |

**Inherited from `Function`**:
`__call__`,
`__getitem__`,
`__setitem__`,
`findzero`,
`numhess`

**Inherited from `object`**:
`__delattr__`,
`__getattribute__`,
`__hash__`,
`__new__`,
`__reduce__`,
`__reduce_ex__`,
`__repr__`,
`__setattr__`,
`__str__`


|  |  |
| --- | --- |
| Properties | [hide private] |

**Inherited from `object`**:
`__class__`


|  |  |
| --- | --- |
| Method Details | [hide private] |

|  |  |
| --- | --- |
| \_\_init\_\_(self, F, period=1)  *(Constructor)* | source code |

x.\_\_init\_\_(...) initializes x; see x.\_\_class\_\_.\_\_doc\_\_ for
signature

Overrides: object.\_\_init\_\_: *(inherited documentation)*

|  |  |
| --- | --- |
| diff(self, X, ind=None) | source code |

Overrides: Function.diff

  


| Home | Trees | Indices | Help | PyDSTool |
| --- | --- | --- | --- | --- |

|  |  |
| --- | --- |
| Generated by Epydoc 3.0.1 on Fri May 4 15:24:07 2012 | http://epydoc.sourceforge.net |
